# The Association of CCL4 rs1634507 and rs10491121 Polymorphisms with Susceptibility of Oral Squamous Cell Carcinoma in an Iranian Population: A Case-Control Study

**DOI:** 10.30699/IJP.2022.538948.2725

**Published:** 2022-02-20

**Authors:** Hamideh Kadeh, Mohammad Eyni, Maliheh Parsasefat, Ebrahim Miri-Moghaddam

**Affiliations:** 1 *Oral and Dental Disease Research Center, Department of Oral and Maxillofacial Pathology, Faculty of Dentistry, Zahedan University of Medical Sciences, Zahedan, Iran*; 2 *Student Research Committee, Department of Hematology and Blood Banking, School of Paramedical Sciences, Birjand University of Medical Sciences, Birjand, Iran*; 3 *Department of Molecular Medicine, Cardiovascular Diseases Research Center, Faculty of Medicine, Birjand University of Medical Sciences, Birjand, Iran*

**Keywords:** ccl4, Gene polymorphism, Oral squamous cell carcinoma

## Abstract

**Background & Objective:**

CCL4 (C-C chemokine ligand4) is a chemoattractant involved in tumors' development, progression, and metastasis. The relationship between the *ccl4* gene polymorphisms and the risk of OSCC has not been studied in Iran. This study aimed to identify the effect of *ccl4* gene polymorphism on OSCC susceptibility in the population of Southeastern Iran.

**Methods:**

In this case-control study, a total of 100 participants, 50 patients with OSCC who were referred to the Department of Oral Pathology, Faculty of Dentistry, Zahedan University of Medical Sciences, Iran, and 50 healthy people were included. The DNA was extracted from the tissue blocks of OSCC patients. The rs10491121 and rs1634507 in the *ccl4* gene were evaluated by the tetra-ARMS (Amplification Refractory Mutation System)- PCR technique. Data were analyzed in SPSS (version 21) using the Chi-square and logistic regression test.

**Results:**

CCL4 genotyping showed that AA+AG genotype in rs10491121 and AA+CA genotype in rs1634507 were slightly higher in control than in the case. Still, the risk of OSCC in both polymorphisms was not significantly different. The minor allele (A) in the rs10491121 and rs1634507 polymorphisms were more common in OSCC compared to the control group (OR = 1.2, 95% CI: 0.66 – 2.22, *P*=0.54) (OR = 1.6, 95% CI: 0.85-3.07, *P*=0.15). There was no association between OSCC histopathological grades and CCL4 genotypes at these two sites.

**Conclusion:**

Our results showed no association between *ccl4* gene polymorphism and the risk of oral cancer in the population of Southeastern Iran.

## Introduction

Oral squamous cell carcinoma (OSCC) is the most common head and neck malignancy (1, 2). It is also one of the ten most common cancers worldwide, with an annual incidence of more than 4260000 cases (3). India and Taiwan present with the highest disease rates (5, 6). Despite various therapeutic approaches, OSCC still has a poor prognosis, resulting in high mortality rates among patients (5-7). Individuals' genetic and environmental conditions are two important factors that increase the chances of developing this disease. Tobacco, smoking, alcohol, chronic inflammation, and viral infection are a few environmental factors that play a role in the disease's formation and progression. Changes in tumor suppressor genes and oncogenes are among the genetic changes that make an individual vulnerable to disease (4, 5, 8). A recent research has shown that polymorphisms in genes involved in suppressing the immune system, thrombosis, angiogenesis, and inflammation increase the incidence of this disease in individuals. (3, 4).

Polymorphisms in the genes interleukin-6 (*il6), *interleukin-8* (il8), il10, *resistin* (RETN),* *phosphatase and TENsin homolog deleted on chromosome 10 (pten), tnf, icam1*, and *ccl4* are among the causes that have been associated with increased risk of this disease in various studies. (1, 6, 9). The *ccl4* gene (C-C chemokine ligand4) on chromosome 17(q11-q21) encodes the CCL4 protein (10, 11). Following mitogenic and antigen entry into individual cells, *ccl4* is secreted in inflam-matory environments or compromised tissue, ultimately contributing to the chemical uptake of immune cells such as natural killers and monocyte to the inflammation and injury site (10, 12). The association of *ccl4* with liver carcinoma, rheumatoid arthritis, AIDS, breast and oral cancer has been investigated and confirmed in previous studies (10, 12-16). It is a pro-inflammatory chemokine involved in macrophage recruitment to the site of infection and stimulation of regulatory T cells, and induction of mast cell degranulation. Because of its role in the expression of several integrins in the endothelium, it regulates macrophage motility. Therefore, increased *ccl4* expression increases tumor invasion and migration (17). *ccl4* is a natural ligand for the CCR5 receptor, which competes with the HIV-1 virus in binding to this receptor. Increasing the level of CCL4 reduces the likelihood of the virus. Myeloid-derived stem cells (MDSCs), a heterogeneous population of myeloid cells, also increase T regulatory cells and thus inhibit the immune cell response by releasing various chemokines, including *ccl4*. On the other hand, when inflammatory conditions of CD4 + cells form complex antigen-carrying dendritic cells, *ccl4* release facilitates the interaction of CD4 + cells with CCR5 on the surface of CD8 cells resulting in an effective long-term immune response (18).

Gene expression, protein structure, and disease resistance can all be affected by single-nucleotide polymorphisms (SNP). The detection of SNP in genes associated with the disease can help diagnose the disease as a possible predictor. In Taiwan, the correlation of *ccl4* gene polymorphisms with resistance and clinic-pathological characteristics of OSCC patients was investigated (10). This association of SNPs in genes can vary depending on the population. 

There has not been a study of the relationship between *ccl4* gene polymorphisms and OSCC patients in Iran. This research aimed to evaluate the association of two *ccl4* gene polymorphisms (rs1634507 and rs10491121) with OSCC patients in southeastern Iran.

## Material and Methods

**Study Population**

In this case-control study, a total of 100 partici-pants, including 50 OSCC patients who were referred to the Department of Oral Pathology, Faculty of Dentistry, Zahedan University of Medical Sciences, Iran, from 2015 to 2018 and also 50 healthy samples, were included. Clinical and histopathological data were obtained from the medical records of the patients. An oral and maxillofacial pathologist reviewed slides of OSCC patients for diagnosis confirmation and histo-pathological grading classification. A pathologist cate-gorized all histopathological blocks of OSCC samples into three categories: well-differentiated, moderately differentiated, and poorly differentiated (19). Samples without clinical information and insufficient paraffin-embedded tumor tissue were excluded from the study. Case and control groups were matched by age, gender, and ethnicity. 

**DNA Isolation and Genotyping**

DNA extraction was done from formalin-fixed paraffin-embedded tissue blocks OSCC patients using the RecoverAll™ Total Nucleic Acid Isolation Kit for formalin- or paraformaldehyde-fixed, paraffin-embe-dded (FFPE) tissues (Ambion, Carlsbad, California, USA) according to the manufacturer's instructions. DNA extracted from white blood cells in the control group using sating out methods. Finally, DNA was eluted into distilled water and restored to -20°C.

The *ccl4* single nucleotide polymorphism rs16345-07 and rs10491121 were genotyped using a tetra- amplification refractory mutation system (ARMS). The primer sequences and amplified products have been summarized in Table 1.

**Table 1:** Primer sequences and product size for Rs 10491121 and rs1634507 SNPs genotyping

Variable	Primers sequencing	Product size (bp)
rs10491121	F-outer: 5′-GGTCCAAGAAAATATCCTGAAATC-3′ R-outer: 5′-AGATTTGAATCAAGATTCACCTGAC-3′ F-inner: 5′-ATCCCCTTCCTGAATTAAGTACG-3′ R-inner: 5′-ATTCCACACTCAAAGACTGACTATAGTT-3′	**Outers:240** **Allele G:171** **Allele A:120**
rs1634507	**F-outer: 5′-ACTGTCACACTCTGACTACGGAGCTG-3′** **R-outer: 5′-CATACCCTCACCCTCAGACACAATG-3′** **F-inner: 5′-AAATTCCTGAGAGAGGGATAAAGCCTTAC-3′** **R-inner: 5′-TTTTCTTGACCTCATGAATGCCGT-3′**	**Outers:298** **Allele C:211** **Inner A:140**

**Fig. 1:**
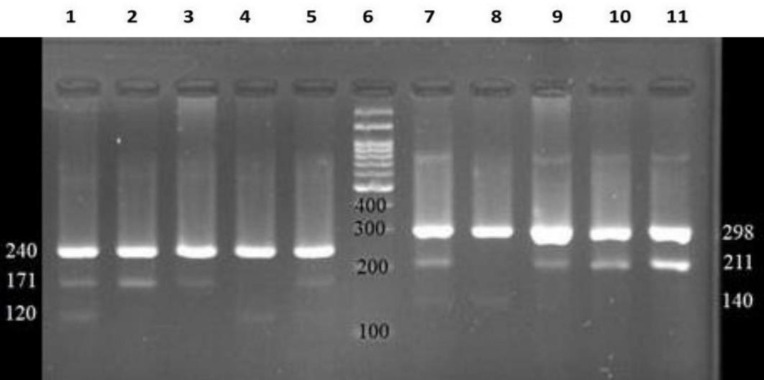
Gel electrophoresis of PCR products of rs10491121 and 1634507 on 2.5 % agarose gel. Wells 1 to 5 show electrophoresis of rs10491121 genotypes (wells 1 and 5; AG, 2 and 3; GG and well 4; AA genotype) and well 6; size marker 100 bp. Wells 7-11 show electrophoresis of 1634507 genotypes (Well 7; AC, Well 8; AA, and 9-11; CC genotype).

Each 20 µL PCR mixture contained genomic DNA (50 ng), 0.1 pmol each of primer (1.5 µL of inner and 0.8 µL of outer primers for rs10491121, 1.7 µL of inner primer, and 0.7 µL of outer primer for rs1634507), and 10 µL of Taq DNA polymerase master mix red (Pishgam, Tehran, Iran), the final volume was reached to 20 µL by adding distilled water. PCR was performed with temperature profile as follows: initial denaturation (95°C for 5 min) followed by 31 cycles of denaturation (95°C for 60s), annealing (59°C for 45s for rs10491121 and 45 s at 62°C in 19 cycles and, 64°C for 45 s in 12 cycles for rs1634507) and extension (72°C for 30 s). At the end of the cycles, a final extension for 72°C at 5 min was performed. The PCR products were electrophoresed on 2% agarose gel and safe stain dye (CinnaGene Iran) (Figure 1), and then visualized using UV light.

**Ethical Considerations**

Patients signed an informed consent form after receiving information about the nature of this study. The protocol was approved by the Ethics Committee of Zahedan University of Medical Sciences (IR.ZAUM-S.REC.1397.393).

**Statistical Analysis**

Demographic, clinical, and laboratory data of patients and controls in association with the results of polymorphism analysis were entered into SPSS software version 21 (SPSS Inc., Chicago, IL., USA). The Chi-square test was used to assess the significant difference of polymorphisms distribution between case and control groups. Odds ratio (OR) was exploited to describe the degree of association between genotypes, and the risk of OSCC development was estimated by logistic regression test. The P-value<0.05 was considered remarkable**.**

## Results

In this study, the *ccl4* gene polymorphism was assessed in 50 OSCC patients, including 17 (34%) males, 33 (66%) females, and 50 control subjects, including 18 (36%) males and 32 (64%) females. As shown in Table 2, the results indicated that the rs10491121 polymorphism (AA+AG) in the case group (50%) was higher than the control group (48%), and the rs1634507 polymorphism (AA+CA) was higher in case (54%) compared to control group (40%). The risk of OSCC is not significant in both of these poly-morphisms. The results were shown that the minor allele (A) in the rs10491121 polymorphism was 32% among OSCC compared to 28% in the control group (OR = 1.2, 95% CI: 0.66 – 2.22, *P*=0.54). Furthermore, the minor allele in the rs1634507 (A) was 30% in the OSCC patient compared to 21% in the control group. In terms of tumor grade, 42% of the OSCC samples were well-differentiated, followed by moderately (40%) and poorly (8%) differentiated. However, there was no correlation between OSCC grade and *ccl4* genotypes (Table 3).

**Table 2:** Odds ratio (OR) and 95% confidence interval (CI) of OSCC (50 cases and 50 control) associated with CCL4 genotypic and allelic frequencies

Variable	Genotypes/Alleles	Case N (%)	Control N (%)	P*-*value	OR (95% CI)
rs10491121	GG	25 (50%)	26 (52%)	-	**1**
	AG	18 (36%)	20 (40%)	0.88	**0.94 (0.4-2.17)**
	AA	7 (14%)	4 (8%)	0.38	**1.8 (0.47-6.99)**
	AA+AG	25 (50%)	24 (48%)	0.84	**1.08 (0.49-2.37)**
	Allele G	68 (68%)	72 (72%)	-	**1**
	Allele A	32 (32%)	28 (28%)	0.54	**1.2 (0.66-2.22)**
rs1634507	CC	23 (46%)	30 (60%)	-	**1**
	AC	24 (48%)	19 (38%)	0.23	**1.65 (0.73-3.71)**
	AA	3 (6%)	1 (2%)	0.25	**3.9 (0.38-40.12)**
	AA+AC	27 (54%)	20 (40%)	0.16	**1.7 (0.80-3.89)**
	Allele C	79 (79%)	70 (70%)	-	**1**
**Allele A**	**21 (21%)**	**30 (30%)**	**0.15**	**1.6 (0.85-3.07)**

**Table 3:** Distribution of CCL4 genotypic in OSCC cases according to histopathological grades

Variable Genotypes		Histopathological Grade			P-value
		W. D^1^ N (%)	M.D^2^ N (%)	P.D^3^ N (%)	
rs10491121	GG	10 (47.6)	12 (60)	3 (33.3)	**0.74**
	GA	8 (38.1)	6 (30)	4 (44.4)	
	AA	3(14.3)	2 (10)	2 (22.2)	
	Total	21 (100)	20 (100)	9 (100)	
rs 1634507	CC	12 (57.1)	8 (40)	3 (33.3)	**0.12**
	AC	8 (38.1)	12 (60)	4 (44.4)	
	AA	1 (4.8)	0 (0)	2 (22.2)	
**Total**	**21 (100)**	**20 (100)**	**9 (100)**

1. Well-Differentiated, 2. Moderately Differentiated, 3. Poorly Differentiated

## Discussion

OSCC is one of the most common cancers globally; despite chemotherapy and radiotherapy, the prognosis is relatively unfavorable, with 5-year overall survival (OS) and disease-free survival estimated to be 47% and 74%, respectively (20-22). Therefore, investigating the disease and discovering the molecules and genes involved in the development and progression of the disease can be helpful in providing targeted therapies and predict the prognosis of the disease (14). According to the previous studies, the incidence of cancer of the lip and oral cavity is 354/864, and the death rate from this cancer is 177/384 people per year (23). 

Genetic and environmental factors are two of the most critical factors in increasing the risk of oral cancer in individuals (5, 10). One of the genes involved in the development and progression of this disease is the *ccl4* gene (10). *ccl4* has been identified as a gene that plays a role in various cancers (17). 

Previous studies have shown that an increase in the expression of *ccl4* contributes to migration and tumor invasion by modulating integrin pathway activation in prostate cancer (24). Inversely, an increase in the expression of *ccl4* in esophageal SCC showed a significant correlation with a favorable prognosis (25). These findings suggest that the importance of *ccl4* expression in cancers is still controversial (10, 26). To the best of our knowledge, limited studies investigate the role of gene polymorphisms of CCL4 in the development of oral cancer (9, 10, 27). In this study, the association between *ccl4* rs 10491121 and rs1634507 polymorphisms and the risk of OSCC in an Iranian population are investigated. We found that the rs1049-1121 polymorphism (AA+AG) and the rs1634507 polymorphism (AA+CA) were slightly higher in OSCC patients than in the control group, but these differences were not significant. This might be because the sample size of our study was small. Other factors, such as race or family history, may affect the result.

Contrary to our study, Lien* et al.* showed that patients with T/T homozygotes of *ccl4* rs1634507 G/T polymorphism were correlated with oral cancer risk rather than patients with G/G homozygotes (10). They suggested that A/G genotype *ccl4* rs10491121 polymorphism employs several T lymphocyte subtypes that increase antitumor immunity and could be a pro-tective factor for oral cancer progression. In addition, they examined the synergistic effect of betel quid and smoking with CCL4 gene polymorphism in OSCC patients. It suggested that tobacco smoking and betel quid induce *ccl4* expression followed by a reinforced inflammatory response, tumor progression, and suppression of antitumor immunity.

Lien* et al.* (9) also found higher *ccl4* expression in OSCC patients compared to the healthy group, and this high level of *ccl4* expression was correlated with higher T and N status in patients with OSCC. Their findings showed that CCL4 increased VEGF-C expre-ssion by downregulation miR-195-3p via the JAK2 and STAT3 signaling pathways in cells of OSCC, and thus, CCL4 could be developed as a therapeutic target for OSCC lymphangiogenesis (9).

Lien* et al.*, in another study, has been reported that CCL4- treated OSCC cells increased expression of angiopoietin-2 (ANGPT2) and angiogenesis in an animal model of OSCC. Therefore CCL4 could serve as a therapeutic target for Inhibiting angiogenesis and metastasis of OSCC (27).

Many studies indicate that *ccl4* plays an essential role in many types of tumors. For example, Wang* et al.* (14) identified the A/G homozygotes of CCL4 rs1049-1121 polymorphism was associated with a lower risk for hepatocellular carcinoma. Another study (28) reported that T/A/A CCL4 haplotype increased the risk of lung cancer in a Chinese population. Hua* et al.* (29) showed that *ccl4* upregulated the VEGF-A expression through activating STAT3, increasing the progression and metastasis of endometrial carcinoma.

It was shown that the high level of *ccl4* expression had induced tumor-associated macrophages (CD163+ cells) infiltration in colon cancer. Also, the levels of VEGF and TNF-α correlated with CCL4 levels in plasma. Therefore levels of chemokines may be a factor for poor prognosis (30). 

## Conclusion

In the present study, the rs10491121 polymorphism (AA+AG) in the OSCC patients was slightly higher (50%) than the control group (48%), and the rs1634507 polymorphism (AA+CA) was higher in OSCC patients (54%) compared to control group (40%). The risk of OSCC is not significant in both of these polymer-phisms. Therefore we found no association between CCL4 SNPs rs1634507 and rs10491121 and the risk of oral cancer in an Iranian population. Due to the limited sample size in our study, further studies with larger sample sizes will be needed to assess the association between CCL4 polymorphisms and OSCC risk.

## Conflict of Interest

None declared.
